# Draft Genome Sequence of *Bradyrhizobium elkanii* Tn*phoA* 33, a Producer of Polyhydroxyalkanoates

**DOI:** 10.1128/genomeA.01502-16

**Published:** 2017-02-23

**Authors:** Erica M. Lopes, Luciano T. Kishi, Camila C. Fernandes, Fernanda Larozza Paganelli, Lucia M. C. Alves, Eliana G. M. Lemos, Jackson A. Marcondes de Souza

**Affiliations:** a Departamento de Tecnologia, Faculdade de Ciências Agrárias e Veterinárias, UNESP–Universidade Estadual Paulista, Jaboticabal, São Paulo, Brazil; b University Medical Center Utrecht, Utrecht, the Netherlands; c Departamento de Biologia Aplicada à Agropecuária, Faculdade de Ciências Agrárias e Veterinárias, UNESP–Universidade Estadual Paulista, Jaboticabal, São Paulo, Brazil; d Faculdade de Ciências Agrárias e Veterinárias, Departamento de Tecnologia, Laboratório Multiusuário de Sequenciamento em Larga Escala e Expressão Gênica, UNESP–Universidade Estadual Paulista, Jaboticabal, São Paulo, Brazil

## Abstract

The genus *Bradyrhizobium* comprises bacteria with the ability to form nitrogen-fixing symbioses with legumes. They are of great interest in agriculture, as well as for the production of biopolymers such as polyhydroxyalkanoates. Here, we report the draft genome assembly of *Bradyrhizobium elkanii* Tn*phoA* 33 comprising 9 Mb, 1,124 contigs, and 9,418 open reading frames.

## GENOME ANNOUNCEMENT

Members of the *Rhizobiaceae* and *Bradyrhizobiaceae* families have the ability to produce degradable biopolymers such as polyhydroxybutyrate (PHB), which can be used in the manufacture of biodegradable plastics (1). Studies based on the genome of the wild-type strain *B. elkanii* SEMIA 587 (2) have shown the potential for the synthesis and accumulation of PHB. The Tn*phoA* transposon was randomly introduced in the genome of *B. elkanii* SEMIA 587, and the production of PHB was evaluated in comparison with the wild type. Cells were lysed for PHB extraction from *B. elkanii* Tn*phoA* 33 (3). Marcondes et al. (4) viewed the expression of *phbC* and *glgA* genes and indicated the synthesis and accumulation of reserves of PHB and glycogen simultaneously. The connection between the storage of PHB and the tricarboxylic acid cycle demonstrates the importance of this polymer in the regulation of carbon balance. PHB probably also plays an important role during symbiosis with plants regarding: (i) energy-reserve offer for cell division during plant infection, (ii) protection for the nitrogenase complex enzyme, (iii) providing the reducing power for maintenance of O_2_ diffusion in the absence of photosynthesis, (iv) recovering bacterial cells after their release from plants to the rhizosphere, and (v) increasing the survival time of the bacteria in the soil rhizosphere (1).

Genomic DNA from *B. elkanii* Tn*phoA* 33 was extracted using a Wizard genomic DNA purification kit (Promega). The library was constructed using the Ion Xpress Plus fragment library kit (Thermo Fisher Scientific), using conditions recommended by the manufacturer. Single-read sequencing (1 × 200 bp) was performed on an Ion Torrent proton sequencer (Thermo Fisher Scientific) on a separate Ion PI chip, according to the manufacturer’s protocols.

The DNA sequencing analysis allowed the partial identification of the *B. elkanii* mutant genome based on 22,962,787 reads sequenced and 18,907,631 reads showing Phred quality scores greater than 20 using PRINSEQ (http://prinseq.sourceforge.net). The clustering of contigs was performed with the SPAdes program (5), resulting in 1,124 contigs (9,542,959 bp), 63.7% GC content, 9,418 annotated open reading frames, and 77 RNAs. Gene prediction and functional annotation of the draft genome were performed using the RAST server (6).

This mutant strain has revealed important genes for (i) the synthesis and accumulation of PHB, mostly *phbA*, *phbB*, and *phbC*, which represent biotechnological application (7); (ii) the processes of nodulation, *nodABC* (8, 9), and biological nitrogen fixation, *nifHDK* and *fixN*; and (iii) the amino acid metabolism, *glgA*, *glnA*, and *glnB* (4), among others. The group of genes described is very important for biopolymer industry and agriculture applications corroborating the versatility of fates for this mutant strain. Finally, this partial genome analysis has shown great similarity to the wild-type genome of *Bradyrhizobium elkanii* hosted in the NCBI databases (2).

### Accession number(s).

The draft genome sequence of *Bradyrhizobium elkanii* Tn*phoA* 33 was deposited at GenBank, NCBI, under accession number MOXO00000000.
